# Effects of *Ginkgo* leaf tablets on the pharmacokinetics of atovastatin in rats

**DOI:** 10.1080/13880209.2019.1622569

**Published:** 2019-06-12

**Authors:** Yan Ren, Haifeng Li, Xing Liu

**Affiliations:** a Department of Pharmacy, The People’s Hospital of Guangrao, Dongying, China;; b Department of Pharmacy, The People’s Hospital of Dongying District, Dongying, China;; c Department of Cardiology, The People’s Hospital of Guangrao, Dongying, China

**Keywords:** Herb-drug interaction, metabolism, coronary heart disease

## Abstract

**Context:** Ginkgo leaf tablets (GLT), an effective traditional Chinese multi-herbal formula, are often combined with atorvastatin calcium (AC) for treating coronary heart disease in clinic.

**Objective:** This study investigated the effects of GLT on the pharmacokinetics of AC and the potential mechanism.

**Materials and methods:** The pharmacokinetics of AC (oral administered at a dose of 1 mg/kg) with or without pre-treatment of GLT (oral administered at a dose of 80 mg/kg/day for 10 days) were investigated in male Sprague–Dawley rats. The effects of GLT on the metabolic stability of AC were also investigated using rat liver microsome incubation systems.

**Results:** The results indicated that the *C_max_*increased from 36.84 ± 4.21 to 48.68 ± 6.35 ng/mL, and the *AUC_(0-t)_* increased from 135.82 ± 21.05 to 77.28 ± 12.92 ng h/mL, and *t_1/2_* also increased from 2.62 ± 0.31 to 3.32 ± 0.57 h when GLT and AC were co-administered. The metabolic stability of AC was also increased (48.2 ± 6.7 *vs.* 36.7 ± 5.3 min) with the pre-treatment of GLT.

**Discussion:** This study indicated that the main components in GLT could accelerate the metabolism of AC in rat liver microsomes and change the pharmacokinetic behaviours of AC. So these results showed that the herb-drug interaction between GLT and AC might occur, and the clinical efficacy could increase when they were co-administered. Therefore, the clinical dose of AC should be decreased when GLT and AC are co-administered.

## Introduction

Atorvastatin, a synthetic and lipophilic statin, inhibits 3-hydroxy-3-methylglutaryl coenzyme A (HMG-CoA) reductase, the rate-limiting enzyme in cholesterol biosynthesis (Dybro et al. 2016; Eng et al. 2016; Cruz-Correa et al. 2017; Dennison et al. 2017). Atorvastatin is commonly prescribed for the treatment of hypercholesterolaemia and for the prevention of coronary heart disease (Kadam et al. 2016; Liu et al. 2016; Bautista et al. 2017; Kasabri et al. 2017; Kashihara et al. 2017). Growing experimental evidence shows the cholesterol-independent pleiotropic effects of this drug, including protection against Alzheimer’s disease, Parkinson’s disease, multiple sclerosis, depression, convulsion, peripheral neuropathy, and intracerebral haemorrhage (Roth et al. 2016; Shu et al. 2016; Wang et al. 2016). Most of the statins except pravastatin are metabolized by the cytochrome P450 (CYP) enzymes (Wei & Zhang 2015; Hsiao et al. 2016). Interactions involving CYP are, therefore, possible. For example, itraconazole has been shown to increase the area under the plasma concentration-time curve (AUC) of atorvastatin acid and lactone by 3- and 4-fold, respectively (Higgins et al. 2014; Dybro et al. 2016).

*Ginkgo* leaf tablet (GLT) is an effective traditional Chinese multi-herbal formula, which is widely used in treating ischaemic cerebrovascular disease in the clinic (Lin et al. 2003; Chung et al. 2006; Yang et al. 2017). The main components of GLT are *Ginkgo* flavone glycosides, ginkgolides, and bilobalides (Liu et al. 2009; Guan et al. 2014; Rao et al. 2014). Atorvastatin calcium (AC) and GLT are often simultaneously used for the treatment of coronary heart disease in China clinics. However, the herb-drug interaction between GLT and AC are still unknown. Therefore, it is essential to investigate the effects of GLT on the pharmacokinetics of AC and its potential mechanism as adverse reactions such as toxicity and treatment failure might occur resulting from concurrent use of herbal drugs with over-the-counter drugs.

In this study, the potential herb-drug interactions of between GLT and AC were systematically investigated. The *in vivo* pharmacokinetics of AC in rats with or without pre-treatment with GLT were investigated using a sensitive and reliable LC-MS/MS method. The effects of GLT on the metabolic stability of AC were also studied using rat liver microsome incubation systems.

## Materials and methods

### Materials and reagents

Standards of AC (purity >98%) were purchased from the National Institute for the Control of Pharmaceutical and Biological Products (Beijing, China). *Ginkgo* leaf tablets were purchased from Sichuan Kelun Pharmaceutical Co., Ltd. (Wang et al. 2016). Pooled rat liver microsomes were purchased from BD Biosciences Discovery Labware (Woburn, MA, USA). Acetonitrile was purchased from Fisher Scientific (Fair Lawn, NJ, USA). Formic acid was purchased from Anaqua Chemicals Supply Inc. Limited (Houston, TX, USA). All other reagents and solvents were of analytical reagent grade.

### Animals

This animal experimental protocol was approved by the Animal Ethics Committee of the Weifang Medical University (Weifang, China). Male Sprague–Dawley rats weighing 220–250 g were supplied by Sino-British Sippr/BK Lab Animal Ltd. (Shanghai, China). The rats were maintained in an air-conditioned animal quarters at 22 ± 2 °C and 50 ± 10% relative humidity. Water and food (laboratory rodent chow, Shanghai, China) were allowed *ad libitum*. The animals were acclimatized to the facilities for 5 days, and then fasted with free access to water for 12 h prior to each experiment.

### Instrumentation and conditions

The determination of AC was performed as previously reported by Sun et al. (2018). The analysis was performed on an Agilent 1290 series liquid chromatography system (Agilent Technologies, Palo Alto, CA, USA). The chromatographic analysis of AC was performed on a Waters X-Bridge C18 column (3.0 × 100 mm, i.d.; 3.5 μm, USA) at room temperature. The mobile phase was water (containing 0.1% formic acid) and acetonitrile (30:70, v:v) at a flow rate of 0.4 mL/min.

The mass scan mode was the positive MRM mode. The precursor ion and product ion were *m/z* 559.2 → 440.2 for AC and *m/z* 419.9 → 199.4 for the simvastatin, respectively. The collision energy for AC and simvastatin were 30 and 25 eV, respectively. The MS/MS conditions were optimized as follows: fragmentor, 140 V; capillary voltage, 4 kV; nozzle voltage, 500 V; nebulizer gas pressure (N_2_), 40 psig; drying gas flow (N_2_), 10 L/min; gas temperature, 350 °C; sheath gas temperature, 400 °C; and sheath gas flow, 11 L/min.

### Pharmacokinetic study

For pharmacokinetic study *in vivo*, 12 rats were equally randomized to two groups, six rats in each group, including AC-only group (1 mg/kg) (A) and AC (1 mg/kg) + GLT (80 mg/kg/day for 10 days) group (B) (Wang et al. 2016; Sun et al. 2018). Group B was pre-treated with GLT solutions by oral gavage at a dose of 80 mg/kg/day for 10 days before the administration of AC, and then AC were orally administered to rats by oral gavage at a dose of 1 mg/kg. Blood samples (0.25 mL) were collected into a heparinized tube via the *oculi chorioideae* vein before drug administration and at 0.083, 0.25, 0.5, 1, 2, 3, 4, 6, 8, 12, and 24 h after drug administration. After centrifuge at 3500 rpm for 5 min, the supernatant was obtained and frozen at −80 °C until analysis.

### Inhibitory effects of GLT on the metabolic stability of AC in rat liver microsomes

Rat liver microsomes were used to determine the metabolic rate of AC. The assay conditions and reaction mixtures were similar as reported previously (Qi et al. 2013; Qin et al. 2014). The reaction mixture was incubated at 37 °C for 5 min and then AC (100 μM) was added. The effects of GLT on the metabolic rate of AC was investigated by adding 350 μg/mL of GLT to rat liver microsomes and preincubating for 30 min at 37 °C, and then AC (100 μM) was added. Aliquots of 30 μL were collected from reaction volumes at 0, 1, 3, 5, 15, 30, and 60 min and 60 μL ice-cold acetonitrile was added to terminate the reaction, and then the sample preparation method was the same as the plasma sample preparation method and determined by LC-MS/MS.

The half-life (*t_1/2_*) *in vitro* was obtained using equation: *t_1/2_* *=0.693/k*.

### Statistical analysis

Experimental values are expressed as mean ± SD. Statistical analysis of results obtained from clinical study was performed using Student’s paired *t-*test. Differences were considered statistically significant when *p* values were <0.05. Statistical analysis was conducted using Graph-Pad Prism version 3.0 for Windows (San Diego, CA, USA).

## Results

### Pharmacokinetic study *in vivo*

**Figure 1.**
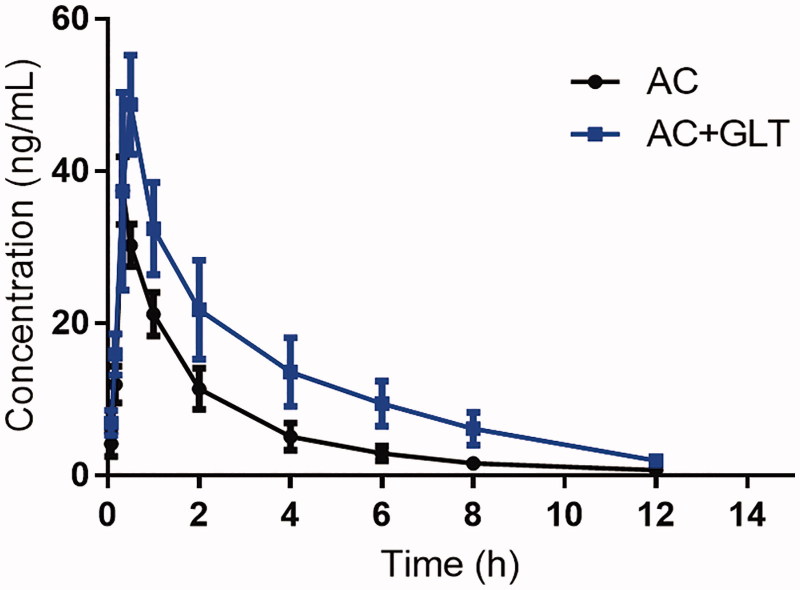
The pharmacokinetic profiles of AC in rats (*n* = 6) after the oral administration of 1 mg/kg AC with or without GLT pre-treatment (80 mg/kg/day for 10 days).

As shown in Table 1 and Figure 1, the results indicated that the *C_max_* (48.68 ± 6.35 *vs.* 36.84 ± 4.21 ng/mL), *AUC_(0-t)_* (77.28 ± 12.92 *vs.* 135.82 ± 21.05 ng h/mL), and *t_1/2_* (3.32 ± 0.57 *vs.* 2.62 ± 0.31 h) increased significantly when GLT and AC were co-administered, which suggested that GLT might influence the pharmacokinetic behaviour of AC when they are co-administered. These results suggested that the herb-drug interaction between AC and GLT might occur when they are co-administered.

**Table 1.** Pharmacokinetic parameters of AC in rats after oral administration of AC (1 mg/kg; *n* = 6, Mean ± S.D.) with or without treatment of GLT (80 mg/kg/day for 10 days).

Parameter	AC	
	AC	AC + GLT
*T_max_ (h)*	0.32 ± 0.03	0.51 ± 0.05*
*C_max_ (ng mL^−1^)*	36.84 ± 4.21	48.68 ± 6.35*
*t_1/2_ (h)*	2.62 ± 0.31	3.32 ± 0.57*
*AUC_(0-t)_ (ng h mL^−1^)*	77.28 ± 12.92	135.82 ± 21.05*
*CL (L h^−1^kg^−1^)*	13.54 ± 2.87	7.65 ± 1.23*

*p < 0.05 indicate significant differences from the control.

### Inhibitory effects of GLT on the metabolic stability of AC with rat liver microsomes

As we know, the metabolism of AC was mainly modulated by CYP3A4 enzymes, and therefore, in this research, the effects of GLT on the metabolic stability of AC were further investigated in rat liver microsomes *in vitro*. The metabolic stability of AC was 36.7 ± 5.3 min, while the metabolic stability was decreased (48.2 ± 6.7 min) in the presence of GLT. The results indicated that the main components in GLT could inhibit the metabolism of AC with rat liver microsomes and change the pharmacokinetic behaviours of AC.

## Discussion

The pharmacokinetic experiments showed that GLT could increase the system exposure of AC in rats when they are co-administered. As the system exposure of AC increased when co-administered with GLT, which suggested that the pharmacological activities of AC might be enhanced. Previous studies have also reported that the GLT could affect the pharmacokinetic profiles of amlodipine, cilostazol, fexofenadine, and clopidogrel when they are co-administered with GLT (Deng et al. 2016; Wang et al. 2016; Turkanovic et al. 2017). The main components in GLT are ginkgolides A, ginkgolides B, bilobalide, quercetin, and kaempferol, and several studies have indicated that the main components in GLT could inhibit the activity of CYP3A4 enzymes (Etheridge et al. 2007; Zadoyan et al. 2012; Palle & Neerati 2017).

To investigate its potential mechanism, the metabolism clearance was also investigated using rat liver microsome incubation systems, and the results revealed that GLT could inhibit its metabolism clearance in rat liver microsomes. AC is metabolized predominantly via CYP3A4 enzyme, and, therefore, co-administration of foods or drugs with influence on CYP3A4 enzymes may affect the pharmacokinetics of AC. Previous studies (Sun et al. 2018) have also reported that drugs or herbs could affect the pharmacokinetics of AC through affecting the activity of CYP3A4 enzyme.

Therefore, this study’s results indicate that when the rats were pre-treated with GLT, the system exposure of AC would be increased significantly. The results suggest that the herb-drug interaction between GLT and AC might occur when they were co-administered. These changes could increase AC efficacy, so it was suggested that the dosage should be adjusted when GLT and AC are co-administered in the clinics.

In conclusion, the results indicated that GLT could influence the pharmacokinetic behaviour AC when they are co-administered. The main ingredients in GLT could inhibit the metabolism of AC in rat liver microsomes, which may be one of the reasons resulting in pharmacokinetic interactions when they were co-administered. Therefore, GLT could increase its clinical efficacy of AC when they are co-administered.

## Data Availability

All data generated or analyzed during this study are included in this published article.
